# Does pre-notification increase questionnaire response rates: a randomised controlled trial nested within a systematic review

**DOI:** 10.1186/s12874-021-01462-z

**Published:** 2021-11-27

**Authors:** Benjamin Woolf, Phil Edwards

**Affiliations:** 1 Department of Psychological Science, University of Bristol, 5 Priory Road, Bristol, UK; 2 Medical Research Council Integrative Epidemiology Unit, University of Bristol, Bristol, UK; 3 Faculty of Epidemiology and Population Health, London School of Hygiene and Tropical Medicine, London, UK

**Keywords:** Pre-notification, Randomised controlled trials, Questionnaire response

## Abstract

**Background:**

Missing outcome data can lead to bias in the results of systematic reviews. One way to address missing outcome data is by requesting the data from the trial authors, but non-response is common. One way to potentially improve response rates is by sending study participants advance communication. During the update of a systematic review examining the effect of pre-notification on response rates, study authors needed to be contacted for further information. This study was nested within the systematic review by randomising authors to receive a notification of the upcoming request for information. The objective was to test if pre-notification increased response rates.

**Methods:**

The participants were study authors included in the systematic review, whose studies were at unclear risk of bias. The intervention was a pre-notification of the request for further information, sent 1 day before the request. The outcome was defined as the proportion of authors who responded to the request for information. Authors were randomised by simple randomisation. Thirty three authors were randomised to the pre-notification arm, and 42 were randomised to the control arm. Authors were blinded to the possibility of an alternative condition.

**Results:**

All authors randomised were analysed. 14/33 (42.4%) authors in the pre-notification arm had returned responses to the questionnaire, and 18/42 (42.9%) in the control arm. There was no evidence of a difference between these groups (absolute difference = − 0.5, 95% CI (− 23.4 to 22.5%), *p* = 1). We received no complaints about receiving the pre-notification.

**Conclusions:**

This study’s results do not support the hypothesis that pre-notification increases response from study authors being contacted for a request for more information. However, the study has a low power, and the results may not generalise to other contexts, methods of administering a pre-notification, or study populations.

**Trial registration:**

Registration and protocol:

This trial is not registered with any trial registry. However, the protocol was posted in advance on the Open Science Framework website and is available on the Open Science Framework website: DOI: 10.17605/OSF.IO/MSV2W or https://osf.io/msv2w/

**Supplementary Information:**

The online version contains supplementary material available at 10.1186/s12874-021-01462-z.

## Introduction

### Background

Missing outcome data is an undesirable feature to have in a study. Missing data will reduce study power and, more worryingly, it also introduces risk of bias, and therefore potentially perturbs a study’s internal validity [1, 2]. Missing outcome data is additionally thought to be a major source of research waste [3, 4]. In a systematic review, specifically, missing information about a study may either contribute to publication bias by leading to a study being excluded from (part of) an analysis, or to a study being assigned an incorrect risk of bias (e.g., unclear instead of low or high).

One potential method for reducing missing data is for the reviewers to contact study authors for more information, for example by sending a standardised request for information form. Among authors whom this form is sent to, missingness will depend on the probability of the author replying to the request for more information. It is therefore important to find ethical ways of reducing non-response to these requests.

To date, we are unaware of any studies specifically addressing how to increase the probability that a study author will reply to a request for more information in a systematic review. However, there is an extensive literature on how to reduce missing outcome data in other settings. One potential method for doing so is notifying participants of the attempt to collect data in advance. This is often termed ‘pre-notification’, ‘pre-contact’, or ‘advanced’ notification or contact. In 2009, Edwards et al. published a systematic review of randomised controlled trials evaluating methods of reducing questionnaire non-response. They found that pre-contact increased response when compared to no pre-contact (OR = 1.5, 95% CI 1.26–1.78, for response after first questionnaire administration, and OR = 1.45, 95% CI 1.29–1.63 for response after final questionnaire administration) [5]. However, this study is now a decade old, so we started an update of this systematic review [6].

A large proportion of studies included in our update did not provide enough information for an unambiguous risk of bias evaluation using the Cochrane Risk of Bias tool, and none of the studies explored this effect in the setting of a systematic review. This study is therefore nested within the author follow up of the aforementioned systematic review and aimed to provide further evidence on the question of whether pre-notification increases response rates to questionnaires specifically in the context of requests for more information in systematic reviews.

### Objectives

To assess the hypothesis that sending study authors an advanced notification of an upcoming request for information, for a systematic review, would increase response rates.

## Methods

### Trial design

This study is a two-arm randomised trial, with participants individually randomised with a 50% chance of the intervention (pre-notification) and control (no pre-notification). There was no change to the trial methods after its commencement. This study followed the CONSORT 2010 guidelines [7]. The protocol was pre-registered on the Open Science Framework at https://osf.io/msv2w/ or DOI 10.17605/OSF.IO/MSV2W .

### Participants

#### Eligibility criteria for participants

Participants were eligible to be entered into the study if they were the corresponding author of a study deemed eligible for a systematic review into the effect of pre-notification on response rates and had provided insufficient detail in the written report for the paper to be judged as high or low risk of bias. In cases in which valid contact details for the corresponding authors were not accessible, other study authors were included in the study instead.

Participants were excluded if no means of email, or other online, communication was found. This was established primarily by checking the stated address in papers. The validity of the address was confirmed by checking the author’s university/personal webpage. In cases of discrepancy the emails were sent to both accounts. If no email could be found Research Gate was checked as another possible means of contact.

### Settings of data collection

The study took place online, using email addresses or Research Gate for the sending of the pre-notification, and the questionnaire, which was itself in Microsoft Word.

### Sample size

No power analysis was conducted to determine the sample size required for this study because the sample size was determined by the number of contactable authors who had provided insufficient information on risk of bias in the systematic review in which this study is nested.

### Randomisation

#### Sequence generation

Participants were assigned using simple randomisation. The intervention and control arm were assigned numerical values (1 or 0 respectively). The first author then used the random number generator on a Casio fx-85GT PLUS calculator to randomly generate a sequence of 0 s and 1 s, each with a 50% probability, to allocate participants to the intervention or control arm.

#### Allocation concealment

Prior to allocation, study authors were pseudoanonymised by physically masking identifiable details. However, after randomisation, authors were unconcealed to send the correct communication to the allocated authors.

#### Implementation

The entire randomisation process was implemented by the first author.

#### Interventions

After randomisation, those participants allocated to the intervention arm received a pre-notification email, Letter 1 in Table 1. All authors were emailed the questionnaire 1 day after the pre-notification email was sent. With the questionnaire, authors in the pre-notification arm were sent Letter 2. All authors in the control arm were sent Letter 3. Follow up contacts were sent at one (Letter 4), and two (Letter 5) weeks after the initial sending of the questionnaire, where a response had not been received by that timepoint. Other than the pre-notification, all communication to the two arms were sent on the same day. The pre-written communication and questionnaire are in Tables 1 and 2.

**Table 1:** Pre-written communication

Letter 1^a^	
Dear [insert name] We emailed you yesterday about your [insert date] paper '[insert title]', which has been included in a partial update to our 2009 Cochrane systematic review into improving response rates to questionnaires. If you are not too busy, we would be very grateful if you could answer the attached questions about the research methods you used? Thank you for taking the time read this email, we look forwards to hearing back from you soon, All the best, Phil Edwards and Benji Woolf	
Letter 2^a^	
Dear [insert name] Thank you very much. I will send you the survey first thing tomorrow. Thank you again, Benji	
Letter 3^a^	
Dear [insert name] Thank you very much for being willing help! Any information would be of use. I will email the questions when I get into to office today. Thank you, Benji	
Letter 4^a^	
Dear [insert name] Thank you very much for being willing help, and sorry for disturbing you from retirement! Any information would be of use. I've attached the questions. Thank you for taking the time to reply/offer help, All the best, Benji	
Letter 5^a^	
Dear [insert name] Thank you very much for your willingness to help us in our review. I have attached the survey to this email. If you are not too busy, we would be very grateful if you could answer the attached questions about the research methods you used. I hope you have a wonderful time on vacation! All the best, and thank you again, Benji	
Letter 6^a^	
Dear [insert name] We emailed you last week with some questions about your [insert date] paper '[insert title]', which has been included in a partial update to our 2009 Cochrane systematic review into improving response rates to questionnaires. If you are not too busy, we would be very grateful if you could answer the attached questions about the research methods you used? Thank you for taking the time read this email, we look forwards to hearing back from you soon, All the best, Phil Edwards and Benji Woolf	
Letter 7^a^	
Dear [insert name] Thank you for taking the time to read this email. We are currently trying to update part of our 2009 Cochrane systematic review into improving response rates to questionnaires, and your [insert date] paper '[insert title]' was selected for inclusion. However, we were hoping you could provide us with some extra information about the methods you used, and would not mind answering some quick questions we will be emailing you tomorrow. Thank you again for taking the time read this email, we look forwards to hearing back from you soon, All the best, Phil Edwards and Benji Woolf	
Letter 8^a^	
Dear [insert name] Thank you for taking the time to read this email. We are currently trying to update part of our 2009 Cochrane systematic review into improving response rates to questionnaires, and your [insert date] paper '[insert title]' was selected for inclusion. If you are not too busy, we would be very grateful if you could answer the attached questions about the research methods you used? Thank you again for taking the time read this email, we look forwards to hearing back from you soon, All the best, Phil Edwards and Benji Woolf	

^a^Email title: Request for further information.

**Table 2:** Questionnaire

In the following boxed please could you give further details about the methods you used to:	
The method used for random sequence generation	
The method used for allocation concealment	
How participants were blinded/masked	
How personnel were blinded/masked	
How outcome assessors were blinded/masked	
The delay between the administration of the pre-notification and the questionnaire	
If you have conducted any other research addressing this question	

### Blinding

#### Blinding of personnel and participants

No active blinding of participants or personnel occurred. However, no material risk of bias should have been introduced. Because participants were unaware of having been randomised, any effect of treatment could not be because of knowing that they had been specially selected for an intervention which others had not received. Although the participant still knew they had received the pre-notification, this knowledge is part of the effect of a pre-notification.

Likewise, although unblinded, because the initial communication was pre-written, study personnel did not have the ability to influence the experience or perceptions of potential participants, and their only means of communication was through a pre-written pro-forma message, with exceptions noted in the Protocol Deviation section.

#### Blinding of outcome assessment

The number of questionnaires in each arm returned was logged unblinded. Data was pseudo anonymised prior to statistical analysis.

### Outcomes

The primary outcome in this paper is the final response rate. This is defined by the proportion of authors which had replied 4 weeks after sending the initial questionnaire, and therefore 2 weeks after the sending of all follow up communication. The secondary outcome is the proportion of authors contacted which had replied at the point at which the first follow-up was sent, 1 week after sending the questionnaire. A reply was defined as any response to the request for more information, and therefore included responses which included missing answers. Both outcomes were assessed after the end of the trial. The primary and secondary outcomes were chosen to mirror the outcomes used in the systematic review.

### Statistical methods

Results were computed by calculating the proportion of responses in allotted times, the absolute and ratio in the proportions in the control and intervention arms, with corresponding 95% confidence intervals, and Chi-Squared test, calculated using the “prop.test” function in R 4.0.2, and the ‘riskratio’ command in the ‘fmsb’ R package [8, 9]. No additional type of analysis was conducted.

## Results

### Protocol deviations

To protocol was written under the assumption that authors would not reply to the pre-notification letter. To not to appear impolite, authors who did reply to the initial pre-notification e-mail were sent modified letters the following day. Specifically, six authors replied to this email on the same day and were sent Letter S1 in Supplementary Table 1, and two replied the following day and were sent Letter S2. Two authors replied to the pre-notification at roughly the same time as the questionnaire were being sent and were sent Letters S3 and S4 respectively instead.

### Participant flow

**Fig. 1:**
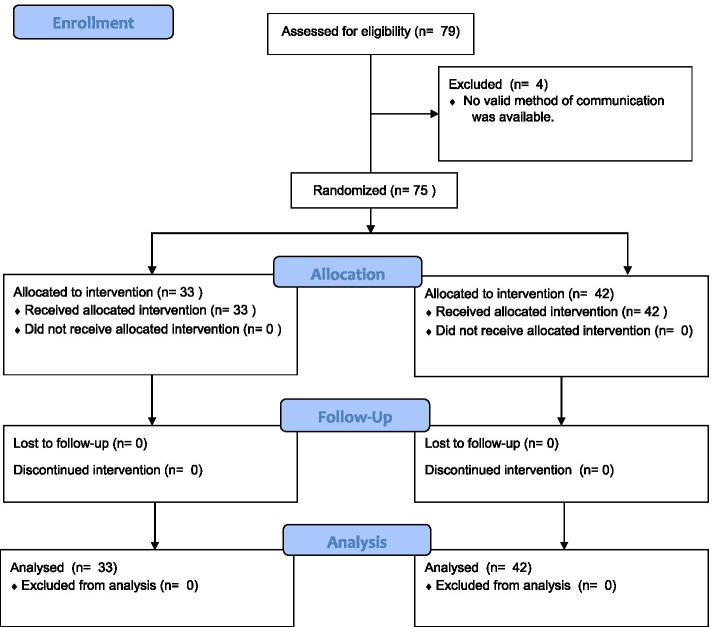
CONSORT flow diagram of participant recruitment

Of the 79 eligible studies, 4 were excluded because no method of communication with any author could be found. In the remaining 75 studies, 67 were corresponding authors, and 8 were other authors. Thirty three were allocated to the pre-notification arm, and 42 were allocated to the control arm (Fig. 1). All participants in the pre-notification arm received the pre-notification 1 day before they received the questionnaire. None of the control arm received the pre-notification. Fifty-six follow-ups were sent after the first week, and 52 were sent after the second week.

### Recruitment

Participants were recruited into this trial implicitly through the request for information. These were sent in early June 2019. As defined in the methods section, participants were then given a month for follow-up. The trial was stopped at the end of this time. Follow-up communication was sent to authors who had not responded 1 week, and 2 weeks after the sending of the initial request for information. Eight authors were contacted through Research Gate, with, by chance, an equal split across arms.

### Baseline data

No baseline data was recorded for this study.

### Numbers analysed

Thirty-three studies are included in the analysis in the pre-notification group, and 42 in the no pre-notification group. Analysis was conducted on an intention-to-treat basis, and included all papers assigned to each intervention.

### Outcomes and estimation

#### Primary outcome

At the end of follow up, 14/33 (42.4%) authors in the pre-notification arm had returned responses to the questionnaire, and 18/42 (42.9%) in the control arm had returned responses to the questionnaire in the no pre-notification condition. The absolute difference between the two arms is − 0.4% (95% CI − 23.0 to 22.1%, Χ^2^ (df = 1) = 4.75 × 10^− 31^, *p* = 1), and the risk ratio is 0.990 (95%CI 0.583 to 1.680, *p* = 0.989).

#### Secondary outcome

Prior to sending the follow up emails, 9/33 (27.5%) authors in the pre-notification condition had returned responses to the questionnaire, and 10/42 (23.8%) in the control arm had returned responses to the questionnaire in the no pre-notification condition. The absolute difference between the two arms is 3.7% (95%CI − 19.2 to 26.1%, Χ^2^ (df = 1) = 5.61 × 10^− 3^, *p* = 0.940), and the risk ratio is 1.145 (95%CI 0.524 to 2.490, *p* = 0.733).

### Ancillary analysis

All authors who returned a questionnaire stated that they had filled them out to the best of the information that they still had available. However, only 7/14 (50.0%) of authors in the pre-notification arm, and 7/18 (38.9%) of authors in the control arm, who returned the questionnaire, provided sufficient information for their studies to be classified as being high or low risk of bias. In addition, only one of the authors sent Letter S3 or S4 returned the questionnaire. After removing these two authors there was still no evidence of a difference between the two groups, absolute difference = − 2.3% (95% CI − 27.1 to 22.7%, Χ^2^ (df = 1) = 5.39 × 10^− 31^, *p* = 1), risk ratio = 0.948 (95%CI 0.549 to 1.635, *p* = 0.848).

### Harms

None of the responses indicated that the prenotification email was not acceptable, and no explicit complaint was received after sending any of the communications, however, one author in the control arm replied “RETIRED” with no other content to the first follow up email.

## Discussion

This randomised trial was nested within a systematic review of randomised controlled trials examining the effect of pre-notification on questionnaire response rates. The trial results imply that pre-notification does not improve the rate of responses to requests for additional information in systematic reviews.

None of the studies included in the review in which this study was nested were examining the effect of pre-notification in the context of requests for information for systematic reviews. This is an important method for determining the quality of studies included in a review and implies an absence of empirical evidence on how to optimise responses in this context. Given the minimal time and cost requirements for conducting this nested trial, we would encourage other systematic reviews to also include a nested study where there is uncertainty over the optimum method. Replications of this specific study may also counterbalance some of this study’s limitations, such as lack of power.

### Limitations

This study has several limitations. Firstly, the width of the 95% confidence intervals for the difference in the response rates is very large. This implies that the null result could be due to low precision, despite the point estimate being very close to the null value. The lack of precision could have been reduced by having a larger sample size, although this was capped due to the pragmatic nature of the inclusion criteria, or by having a more balanced randomisation list.

A second potential limitation is that the intervention used was the same intervention as the one the included study authors’ studies had examined. This may have meant that contacted authors guessed that they were in the intervention or control arm of a randomised controlled trial examining the effect of pre-notification. This occurred for certain in one instance in the intervention arm. If so, then some degree of unblinding would have occurred, which might have biased the results. However, although ultimately unknowable, it seems probable that this would have only occurred for a minority of authors, in which case any bias is likely to be small.

Finally, there is a potential risk of bias from unblinded study personnel. However, because most communication with the participants, prior to responses, was pre-written the magnitude of any bias this could introduce should be small. In addition, the effect remained consistent after removing the two studies where communication deviated from the protocol.

### Interpretation

There is an extensive literature examining the role of pre-notification on response rates. This has generally found that pre-notification is beneficial to response, as summarised in, e.g., Edwards et al. [4]. This is contrary to the results of this study, which did not find evidence for an effect of pre-notification on response rates. This result is also contrary to the overall finding of the update to this review, in which this study was nested.

This could be for three reasons. Firstly, the true estimate might be lower than is typically thought. After removing studies at high or unclear risk of bias, we found that the ratio of the odds of responding given a pre-notification or no pre-notification decreased from OR = 1.38 (95%CI: 1.25–1.53) to OR = 1.11 (95% CI: 1.01–1.21) [5].

Secondly, the result of this study might be due to the small sample size, and thus low power. This is supported by the large confidence intervals for the risk difference. Any issue with power would be exacerbated if the effect estimate is smaller than the one typically used in the literature.

Finally, it may be that pre-notifications are an ineffective intervention in the context of a systematic reviews requests for information from study authors.

### Generalisability

Both the original and updated review found substantive heterogeneity across studies. It is possible that some of this heterogeneity may be because the effect of pre-notification differs depending on the context or population in which it is used. If this the case, then the study’s results might not generalise to other context or study populations, and the averaged effect from the systematic reviews may not be transferable to this setting. Likewise, differences in results might be different depending on the nature of the pre-notification (e.g., delay between sending of pre-notification and questionnaire, method of sending prenotification/questionnaire, etc). Either of these possibilities would limit the generalisability of this study’s results to other settings.

## Conclusion

This randomised controlled trial sought to assess the impact of pre-notification on response to a request for more feedback by study authors, whose studies had been included in a systematic review. The study found no evidence to support a difference in response across the control and intervention group in this context. This is probably due to either low power to detect a plausible effect size, or the absence of an effect of pre-notification in the context of request for future information in systematic reviews. Future replications by additional randomised controlled trials embedded within the request for information of systematic reviews is required to definitively determine the effectiveness of this intervention in this context.

## Supplementary Information

**Additional file 1: Supplementary Table 1** Description of emails which deviated from the protocol.

## Data Availability

Data and materials not available in the paper will be made available through contacting the corresponding author.
