# Supplementary material for: Does pre-notification increase questionnaire response rates: a randomised controlled trial nested within a systematic review
Source: BMC Med Res Methodol. 2021 Nov 27;21:259. doi: 10.1186/s12874-021-01462-z (PMC8627620; doi:10.1186/s12874-021-01462-z)
Supplement: Supplementary file 1 — Additional file 1: Supplementary Table 1 Description of emails which deviated from the protocol. [file 12874_2021_1462_MOESM1_ESM.docx]

| Letter S1* |
| --- |
| Dear [insert name]  Thank you very much. I will send you the survey first thing tomorrow.  Thank you again,  Benji |
| Letter S2* |
| Dear [insert name]  Thank you very much for being willing help! Any information would be of use. I will email the questions when I get into to office today.  Thank you,  Benji |
| Letter S3* |
| Dear [insert name]  Thank you very much for being willing help, and sorry for disturbing you from retirement! Any information would be of use. I've attached the questions.  Thank you for taking the time to reply/offer help,  All the best,  Benji |
| Letter S4* |
| Dear [insert name]  Thank you very much for your willingness to help us in our review. I have attached the survey to this email. If you are not too busy, we would be very grateful if you could answer the attached questions about the research methods you used. I hope you have a wonderful time on vacation!  All the best, and thank you again,  Benji |

Supplementary Table 1: Description of emails which deviated from the protocol.
